# Search for Hydrophilic Marine Fungal Metabolites: A Rational Approach for Their Production and Extraction in a Bioactivity Screening Context

**DOI:** 10.3390/md9010082

**Published:** 2011-01-10

**Authors:** Carine Le Ker, Karina-Ethel Petit, Jean-François Biard, Joël Fleurence

**Affiliations:** University of Nantes, Faculty of Pharmacy, MMS–EA 2160, F-44000 Nantes, France; E-Mails: carine.le-ker@etu.univ-nantes.fr (C.L.K.); karina.petit@univ-nantes.fr (K.-E.P.); jean-francois.biard@univ-nantes.fr (J.-F.B.)

**Keywords:** marine fungi, hydrophilic metabolites, bioactivity, agar surface fermentation, submerged fermentation, extraction process

## Abstract

In the search for bioactive natural products, our lab screens hydrophobic extracts from marine fungal strains. While hydrophilic active substances were recently identified from marine macro-organisms, there was a lack of reported metabolites in the marine fungi area. As such, we decided to develop a general procedure for screening of hydrophobic metabolites. The aim of this study was to compare different processes of fermentation and extraction, using six representative marine fungal strains, in order to define the optimized method for production. The parameters studied were (a) which polar solvent to select, (b) which fermentation method to choose between solid and liquid cultures, (c) which raw material, the mycelium or its medium, to extract and (d) which extraction process to apply. The biochemical analysis and biological evaluations of obtained extracts led to the conclusion that the culture of marine fungi by agar surface fermentation followed by the separate extraction of the mycelium and its medium by a cryo-crushing and an enzymatic digestion with agarase, respectively, was the best procedure when screening for hydrophilic bioactive metabolites. During this development, several bioactivities were detected, confirming the potential of hydrophilic crude extracts in the search for bioactive natural products.

## 1. Introduction

Marine fungi are efficient producers of organic molecules with original chemical skeletons and various associated bioactivities [1,2]. However, a majority produce hydrophobic compounds isolated, through organic solvent extraction and purification [3]. Nevertheless, some results have proved that bioactive hydrophilic substances can be obtained from marine fungi [4–6]. In the same way, promising hydrophilic pharmaceutical substances have been isolated from other marine organisms [7] or from terrestrial micro and macro fungi [8]. This fact prompted us to broaden our bioactivity screening method to the search for hydrophilic fungal metabolites.

According to the literature, submerged fermentation (SmF) followed by extraction of the metabolites from the filtrate [4–6,8] is the most current procedure, probably due to the easy accessibility of solubilized compounds in the culture filtrate. However, some disadvantages are apparent. Firstly, fungal products not excreted are lost, and these types of products are often chemically different from those excreted. Secondly, fungi are known to adapt their metabolic pathways according to their environment [9], so they could produce a different variety of metabolites in other cultural conditions, such as in solid state fermentation (SSF) like agar surface fermentation (ASF).

The aims of the present work are: firstly, to develop a mode of production of bioactive hydrophilic metabolites by fungi in order to obtain large biomasses with the maximum amount and variety of metabolites, and, secondly, to perform an extraction process so as to retrieve the maximum quantity of biosynthesized metabolites, without any degradation during the process.

To achieve these objectives, several issues need to be resolved: which polar solvent (methanol or water) to select; which fermentation process (SmF or ASF) to use; which kind of raw material (mycelium or medium) to extract; which kind of extraction to apply so as to recover both intracellular metabolites from the mycelium and excreted metabolites trapped within agar-agar.

The proposed method will take into account the difficulties inherent in handling hydrophilic substances [10] and be compatible with the constraints of a bioactivity screening, *i.e.*, easily applicable to a large number of strains for routine use at the laboratory scale.

Before starting the experiments, some choices were made regarding the objectives described above. We decided to work on six different marine fungal strains as a compromise between the minimum necessary to draw a general conclusion from the results and the maximum achievable given our analysis capacity. All were chosen from our marine fungal collection for their affiliation to various genera and their potential as producers of bioactive hydrophobic substances.

Among several different fermentation methods, SmF and ASF were selected to investigate the bioactive metabolite production.

Although some methods have been described for the extraction of hydrophilic extracellular metabolites from SSF [11], none deal with the problem of the solubilization of the metabolites trapped in a gelled solid support of agar-agar. We decided to test two solvents, methanol and water, on mycelium and medium (agar-agar).

Methanol can dissociate fungal walls and extract the metabolites trapped within agar-agar. However, this polar solvent is not efficient for the extraction of macromolecules and could destroy sensitive macromolecules. Moreover, this method is restricted to solid medium.

Water is more effective to extract hydrophilic compounds, however, a complementary method, such as a mechanical or enzymatic one, is required. We evaluated the efficiency of cryo-crushing and enzymatic digestion for both mycelium and medium (agar-agar).

Once the fermentation method and the extraction process had been determined, it became important to choose the raw material to extract. Mycelium and medium were extracted separately and compared. Although it increases the number of experiments, the advantage of dealing with the mycelium and medium separately lies in producing less complex crude extracts enriched in the target compounds.

In order to interpret the results, biochemical analyses and biological assays were used. We chose quantitative assays of proteins and sugars as criteria for the detection of hydrophilic metabolites, considering that a large production of primary metabolites may be predictive of a large production of secondary metabolites. A specific neuroactivity assay on blowfly larvae and a commonly used cytotoxicity assay on KB cells were used to monitor the presence of bioactivity.

**Figure 1:**
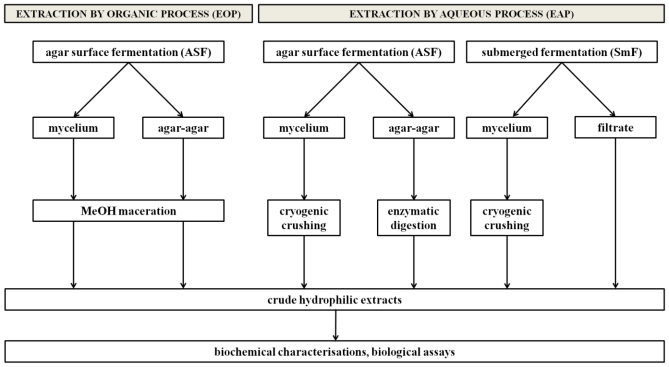
Protocols developed for the optimization of an extraction model for screening hydrophilic fungal metabolites.

Figure 1 shows the general protocol used in this study. All conditions were tested in triplicate.

## 2. Results and Discussion

### 2.1. Choice of the extraction process: organic or aqueous process?

Biochemical analyses (Table 1) on ASF showed that extraction by the aqueous process (EAP) was more efficient than by the organic process (EOP). Compared with EOP, protein yields from EAP were greater by a factor of about five for mycelium (14.19 mg/g dw *vs.* 2.75 mg/g dw) and agar-agar (0.36 mg/mL *vs.* 0.08 mg/mL) crude extracts. Similarly, differences in sugar yields were also obtained between crude extracts from EAP and EOP (mycelium: 44.18 mg/g dw *vs.* 23.33 mg/g dw; medium: 2.21 mg/mL *vs.* 0.38 mg/mL). Such dissimilarities between the two extraction processes can be explained by the lower solubility of hydrophilic compounds in methanol.

Concerning the diversity of proteins found in these crude extracts, complex electrophoretic patterns were observed over a wide range of molecular weights from 3.5 to 250 kDa (data not shown). However, more bands were recognized in cryo-crushed mycelium and digested agar-agar EAP extracts, confirming that EAP is more efficient quantitatively and qualitatively for recovering macromolecules.

According to Table 2, neuroactivity was observed more frequently in EOP extracts (10 active extracts *vs.* 6 active extracts) but the intensity was higher in EAP extracts (minimal effective dose MED: 12.5 mg/mL *vs.* 50 mg/mL). In the same culture conditions, EAP was more efficient at extracting the hydrophilic cytotoxic compounds than EOP (5 *vs.* 0 cytotoxic extracts). However, in all EAP extracts, values of IC_50_ were weak according to international criteria [12].

In conclusion, EAP was more efficient in the extraction of fungal hydrophilic metabolites, for mycelium as well as for medium, regarding biochemical and biological assays. This extraction process was selected for our bioactivity screening model despite being more difficult to implement.

### 2.2. Choice of the fermentation method: SmF or ASF?

On average, the amounts of biomass produced per liter of culture medium by the six studied strains in ASF were three-times higher than those produced in SmF (Table 3). However, *Penicillium citreonigrum* and *Pycnidiophora dispersa* strains gave more comparable amounts of biomass (ASF: 50.0 and 54.2 g/L; SmF: 35.6 and 31.7 g/L, respectively). These two strains grew differently from the others: the mycelium developed on the surface of the liquid medium, mimicking growth in ASF conditions and so explaining the comparable amounts of biomass.

EAP was chosen so that both types of fermentation could be compared.

**Figure 2:**
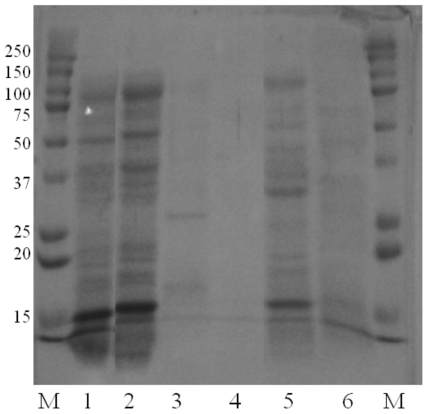
Analysis of protein components of EAP extracts from *P. citreonigrum* strain in ASF (agar surface fermentation) and SmF (submerged fermentation). SDS-PAGE electrophoresis (12% acrylamide) and successive Coomassie blue and silver stainings. M: molecular weight markers (in kDa), Lanes 1: cryo-crushed mycelium from ASF, 2: cryo-crushed mycelium from SmF, 3: digested agar-agar from ASF, 4: inert agar-agar, 5: culture filtrate from SmF, 6: inert culture filtrate.

Both fermentation methods gave comparable electrophoretic patterns (Figure 2, e.g., *P. citreonigrum* extracts, mycelium: lanes 1 and 2, medium: lanes 3 and 5).

Similar protein and sugar yields (14.19 mg/g dw *vs.* 14.27 mg/g dw and 44.18 mg/g dw *vs.* 43.36 mg/g dw respectively) were observed on average in mycelium crude extracts from ASF and SmF (Table 1). However, the amount of proteins and sugars in the medium were greater in ASF than SmF, suggesting that the destination of some macromolecules was affected by the culture conditions (proteins: 0.36 mg/mL *vs.* 0.25 mg/mL and sugars: 2.21 mg/mL *vs.* 2.12 mg/mL). This difference is in agreement with previous works on secreted fungal proteins [13,14].

As crude extracts from both fermentations were quantitatively and qualitatively comparable, the bioactivities were investigated. The bioactive compounds detected are synthesized by the same species cultivated in ASF or SmF (Table 2). However, ASF seemed more suitable to promote the production of neuroactive compounds than SmF: 6 active extracts *vs.* 4 active extracts; highest neuroactivities observed (e.g., *P. citreonigrum*: MED of 12.5 mg/mL for mycelium crude extracts from ASF *vs.* 50 mg/mL for mycelium crude extracts from SmF). As has already been demonstrated for the production of enzymes, lipids or hydrophobic metabolites [14–16], neuroactive hydrophilic metabolite production by fungi is dependent on the fermentation used and these molecules are intracellular.

No marked difference was observed concerning the cytotoxicity: 5 active extracts and similar IC_50_ for both types of fermentation (Table 2). Either fermentation method is suitable to produce cytotoxic substances.

As ASF produces greater quantities of biomass and promotes hydrophilic neuroactive metabolite production, this fermentation method would be preferred in a bioactivity screening context for the simultaneous detection of neuroactive and cytotoxic hydrophilic compounds in extracts. At the laboratory scale, the use of ASF rather than SmF brings significant benefits: a lower risk of microbial contamination, lower sterility demands, no enzymatic degradation, smaller volume of water, less cost and energy [17]. Moreover, ASF can be used on smaller quantities of culture medium in a shorter time than SmF, indicating its potential for profitable cultures on a large scale.

### 2.3. Choice of the raw material to extract: mycelium and/or medium?

EAP was chosen so that both raw materials could be compared.

We investigated both mycelium and medium extracts in order to prove the greater interest of one over the other. In all cases, mycelium extracts were richer in proteins and sugars compared to those of agar-agar (Table 1).

Mycelium and medium extracts did not exhibit the same electrophoretic patterns (Figure 2, lanes 1 and 2: mycelium from ASF and SmF respectively; lanes 3 and 5: medium from ASF and SmF respectively), even though some bands were observed in both extracts.

Concerning the neuroactivity in ASF conditions, mycelium extracts were more active than agar-agar extracts: 4 *vs.* 2 active extracts and MED of 12.5 mg/mL *vs.* 100 mg/mL (e.g., *P. citreonigrum*, Table 2). Similar results were observed in SmF extracts. Neuroactive hydrophilic substances did not seem to be excreted by fungi into the medium or only in small quantities. These results demonstrate the advantage of working on intracellular bioactive fungal metabolites, neglected until now. For purposes of neuroactivity screening, mycelium seems to be more attractive than medium and, thus, should be independently investigated.

Cytotoxicity was detected in both mycelium and medium extracts from ASF and SmF with no marked differences (e.g., *Phoma* sp.) or in either mycelium extracts (*Pycnidiophora dispersa*) or medium extracts (*Phoma exigua* and *Scopulariopsis* sp.)(Table 2). These observations revealed the diversity of cytotoxic compounds in our six studied strains and led us to conclude that mycelium and medium should be independently investigated in a cytotoxicity screening context.

### 2.4. The proposed strategy

**Figure 3:**
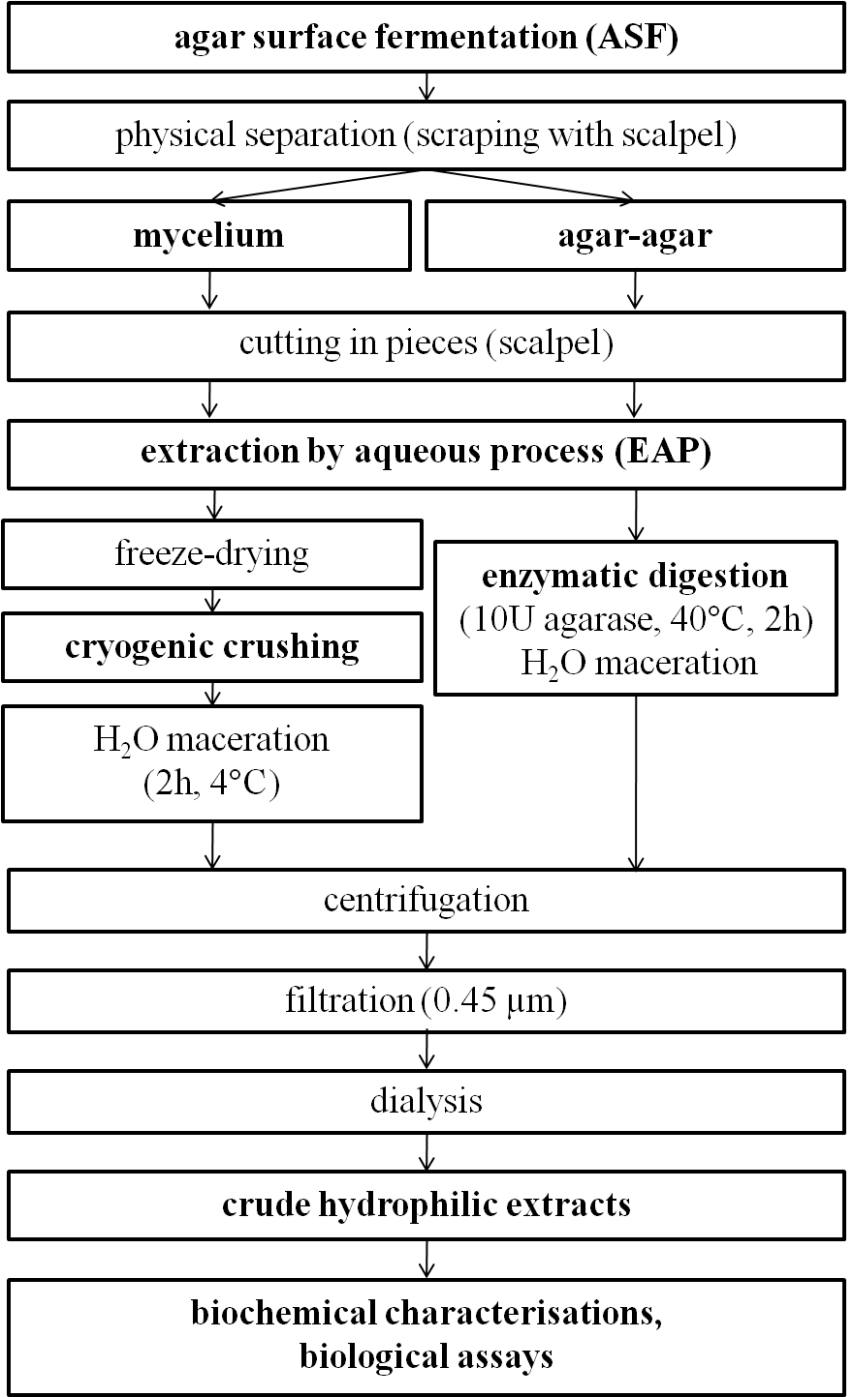
Proposed protocol for the production and extraction of bioactive hydrophilic fungal metabolites.

The objective of this study was to build a rational methodology for the production and extraction of hydrophilic bioactive fungal metabolites. The outcome is that fungal production by ASF and extraction by an aqueous process using cryo-crushing of mycelium and enzymatic digestion of medium with agarase is the best strategy to screen bioactive hydrophilic substances. The resulting general procedure is presented in Figure 3.

According to this protocol and to the bioactivity results, some selected crude extracts were purified. A process of partial-purification by ammonium sulfate (AS) and trichloroacetic acid (TCA) enabled the protein and peptide fractions to be concentrated (data not shown). The use of TCA could be justified, with regard to the AS action, by its efficiency in precipitating very diluted proteins, as in the case of metabolites secreted into the medium [18]. Bioactivities increased with fractionation, proving that this technique is suitable for further purifications (data not shown).

### 2.5. Marine fungi, a promising source of bioactive hydrophilic substances

This study highlighted the production of bioactive hydrophilic substances by marine fungi, hitherto neglected. In fact, although only six marine fungal strains were screened for the detection of hydrophilic neuroactive and cytotoxic metabolites, several of the obtained extracts exhibited bioactivities. Identification of the bioactive compounds should be investigated. The probability of finding some molecules already described is low because no neuroactive hydrophilic substances have yet been identified in fungi. However, hydrophilic protein phytotoxins have already been described in terrestrial *Phoma* strains [19–21]. The presence of such toxins in *Phoma* extracts could explain the observed neuroactivity and, thus, need to be investigated.

Similarly, no cytotoxic hydrophilic compounds have previously been reported for the genera *Pycnidiophora*, *Scopulariopsis* and *Phoma*. Thus, the activities observed in mycelium extracts of these strains could reveal the presence of undescribed hydrophilic compounds. Some cytotoxic hydrophilic proteins, such as ribotoxins [22] and lectins [8,23] have already been described from *Aspergillus*, *Fusarium* and *Rhizoctonia* strains. They have more recently been identified in various genera [24,25] but have not yet been mentioned in the species studied here.

This study has demonstrated the production of potentially bioactive fungal metabolites by marine strains. These findings confirm the need to investigate marine fungi for their production of hydrophilic substances in the search for new natural molecules of pharmaceutical interest.

## 3. Experimental Section

### 3.1. Fungal strains and culture medium

All studied strains were isolated from samples collected in shellfish-farming areas of the Loire estuary on the French Atlantic coast. Fungal strains were identified by sequencing the internal transcribed spacers (ITS 1 and 2) and beta-tubulin regions of the ribosomal RNA cluster as *Penicillium citreonigrum*, *Phoma exigua* var *exigua*, *Phoma* sp., *Scopulariopsis* sp., *Pycnidiophora dispersa* and *Chrysosporium queenslandicum*. All strains were maintained as stock cultures on Dextro Casein Agar (DCA) tubes (dextrose 40 g, enzymatic digest of casein 10 g, agar 15 g, natural filtered seawater 1 L), stored under a paraffin oil layer and conserved in our fungal collection under the reference numbers MMS 029, MMS 719, MMS 797, MMS 850, MMS 940 and MMS 946, respectively (MMS: Marine Fungal Collection, University of Nantes). For the current work, the six fungal strains were cultured on yeast extract sucrose agar medium (YES) in two types of fermentation: SmF and ASF in the culture conditions commonly used in our laboratory (27 °C, natural light). This medium was chosen for its high concentration in sugar and amino acids in order to promote fungal growth, metabolite production and glycosylation mechanisms. Yeast extract medium was composed of 20 g yeast extract, 150 g saccharose, 0.5 g MgSO_4_, 7H_2_O, 0.01 g ZnSO_4_, 7H_2_O and 0.005 g CuSO_4,_ 5H_2_O dissolved in 1 L of natural filtered seawater and was supplemented with 20 g of agar for ASF.

### 3.2. Agar surface fermentation: culture and extraction

#### 3.2.1. Agar surface fermentation

Agar surface fermentations were performed in Petri dishes (16 cm) containing 80 mL of YES agar medium. Media were inoculated with fungal conidia of the six different strains taken from stock cultures. Each Petri dish was then incubated at 27 °C, under natural light, until the dish surface was totally covered by the mycelium, the incubation time corresponding to the required growth time (15 to 45 days). Mycelia were then separated from their media by scraping them from agar-agar with a scalpel. This mechanical separation was selected for its simplicity and its easy application on a large quantity of fungal cultures at the laboratory scale. Then mycelia were washed several times with distilled water and both mycelia and agar-agar were cut into small pieces using a scalpel.

#### 3.2.2. Extraction of agar-agar

##### 3.2.2.1 Extraction of agar-agar by aqueous process (EAP)

A preliminary study had been developed on artificial agar-agar supplemented with standards (albumins and insulin): a cryo-crushing followed by a water maceration had been compared to an enzymatic digestion with agarase at several units. The outcome from this preliminary study was that an enzymatic digestion was more efficient and easier to achieve; moreover 10 U of agarase was sufficient to liquefy the agar-agar completely in a short time (2 hours).

Thus, pieces of agar-agar from ASF were suspended in distilled water containing 10 U of agarase from *Pseudomonas atlantica* (Sigma A-6306, USA) solubilized in distilled water and the homogenates were stirred at 40 °C for 2 h (one unit of agarase is expressed as the amount of enzyme producing 1 μg of agar per minute at 40 °C). Once the agar-agar was liquefied, enzymatic digestion was stopped by cooling and the agar-agar residues were separated from solubilized compounds by centrifugation (15,000 g for 30 min, then 20,000 g for 15 min at 4 °C). Supernatants were collected to constitute crude extracts of extracellular hydrophilic fungal metabolites and filtered under vacuum to remove spores (0.45 μm PTFE membrane filters, Sartorius, Germany). As they are undesirable for the effective operation of many analyses, salts were removed from crude extracts by dialysis, using cellulose tube (MW cut-off 3,500 Da, cellu.sep®, USA) against distilled water under shaking at 4 °C.

##### 3.2.2.2. Extraction of agar-agar by organic process (EOP)

Metabolites contained in pieces of agar-agar were extracted by a shaken methanolic maceration at room temperature. Agar-agar residues were eliminated by successive filtrations, and liquid organic phases were filtered under vacuum to remove spores (0.45 μm) then evaporated to obtain dry extracts. Hydrophobic compounds were removed by a liquid/liquid partitioning using CH_2_Cl_2_/H_2_O (3/1, v/v), and aqueous phases containing the target compounds were kept as the extracellular hydrophilic crude extracts. Salts were removed by dialysis as described above.

#### 3.2.3. Extraction of mycelium

As for agar-agar, the hydrophilic metabolites contained in mycelium pieces from ASF were extracted by two processes: an organic process (EOP) consisting of a methanolic maceration as described above and an aqueous process (EAP) consisting of a cryo-crushing followed by a distilled-water maceration. Concerning the latter, pieces of mycelium were freeze-dried, weighed and then ground into powder in liquid nitrogen. Powders were immersed in distilled water at 4 °C under shaking for 2 h. Mycelia extraction residues were removed by a gauze filtration. Macerates were centrifuged (10,000 g, 20 min, 4 °C) and supernatants were collected to constitute crude extracts of intracellular hydrophilic fungal metabolites. Both supernatants and aqueous phases containing target compounds were kept as the hydrophilic mycelium crude extracts. Crude extracts were finally filtered under vacuum to remove spores (0.45 μm) and then dialysed as described above.

Concerning the extraction of mycelium by an aqueous process, an enzymatic digestion could not be considered because fungal walls are species-dependent, with a high level of complexity and variability [26]. Thus, cryo-crushing was the only technique used.

### 3.3. Submerged fermentation: culture and extraction

#### 3.3.1. Submerged fermentation

Submerged fermentations were performed in 500 mL Erlenmeyer flasks with 250 mL of YES medium. A suspension of 0.5 × 10^6^ spores/mL was prepared from ASF and inoculated. Cultures were then incubated at 27 °C under natural light (25 to 50 days). A paper filtration under vacuum separated the mycelia from the culture media. Then mycelia were washed several times with distilled water and cut into small pieces using a scalpel.

#### 3.3.2. Extraction from SmF

Only one extraction process, EAP, was applied on mycelia from SmF. Mycelium pieces were cryo-crushed in liquid nitrogen and macerated in water as described above for mycelia from ASF. Both culture filtrates and mycelium macerates were centrifuged (10,000 g, 20 min, 4 °C). Supernatants were collected to constitute crude extracts of intracellular and extracellular hydrophilic fungal metabolites. Finally, crude extracts were filtered under vacuum to remove spores (0.45 μm) and dialysed.

### 3.4. Biochemical and bioactivity evaluations

#### 3.4.1. Characterization and measurement

Total protein contents of extracts were determined according to the Bradford method [27] with bovine serum albumin as a standard. Characterization of proteins was performed by sodium dodecyl sulfate polyacrylamide gel electrophoresis according to the method of Laemmli [28] using a 12% resolving gel, a 5% stacking gel and Precision Plus Protein from Bio-Rad as a molecular weight marker. Proteins and peptides were stained with Coomassie blue (Coomassie Brilliant Blue 5R 250, Sigma), periodic acid Schiff (Sigma-Aldrich) and silver nitrate (Silver Stain Plus, Bio-Rad).

Total sugars were estimated using the colorimetric phenol-sulfuric acid method [29] with L-glucose as a standard, and expressed as mg of glucose per g of dried weight. In brief, 1 mL of sample solution was vortexed with 1 mL of 5% phenol in water before adding 5 mL of concentrated sulfuric acid rapidly from a glass dispenser. After standing for 30 min at room temperature, the absorbance of the sample solution was measured at 485 nm against a blank. In addition, estimations of proteins and sugars were made on inert YES solid and liquid media extracted like those from fungal fermentations.

#### 3.4.2. Bioactivity assays

##### 3.4.2.1. Neuroactivity assay on blowfly larvae

The neuroactivity of extracts was evaluated following the method described by Zoltkin [30]. All assays were performed with larvae of the blue fly *Calliphora vomitoria* which were at the last stage before pupariation. The larvae were obtained from fishing supply stores. Extracts to be tested were dissolved in distilled water at an initial concentration of 100 mg/mL. Test solutions were injected (0.1 μL per mg of larval body) using a 10 μL micro-syringe with a thin hypodermic needle (Hamilton, Bonaduz, Switzerland). The needle was inserted into the ventral side at the level of the last abdominal segment and extract was slowly injected. The time (T) required for immobilization of the larvae after injection was measured. If no immobilization occurred after 10 min, the test was considered negative. Three replicates were tested for each dose and the median value was calculated. If a positive response was observed at the initial tested concentration, a range of dilutions was prepared and tested. The Minimal Effective Dose (MED) is defined as the lowest concentration producing an immobilization of larvae. A crude extract was considered interesting if it exhibited a maximal MED of 25 mg/mL (internal criteria according to our own experiments). Domoic acid (0.125 mg/mL) was used as a positive control [31] and distilled water as a negative control.

##### 3.4.2.2. Cytotoxicity assay

The epidermoid human carcinoma KB cells (ATCC CCL 17, Rockville, MD) were maintained at 37 °C in 5% CO_2_ with Eagle’s basal medium (BME) supplemented with 10% (v/v) fetal calf serum, 1% (v/v) glutamine 200 mM and 1% (v/v) streptomycin/penicillin 10 mg/mL. 50 μL of a 2 × 10^5^ cell/mL suspension was placed in wells of 96-well culture plates and incubated for 48 h. Serial dilutions of extracts in BME were added (initial concentration: 250 μg/mL). Microplates were incubated for 72 h. Then 3-(4,5-dimethyl-2-thiazolyl)-2,5-diphenyl-2H-tetrazolium bromide (MTT, purchased from Sigma-Aldrich, 5 mg/mL in distilled water) was added. After 2 h of incubation, dyed formazan crystals were dissolved in isopropanol 0.04% HCl 1N and absorbance values were measured at 570 nm and 630 nm (background noises) using a scanning multi-well spectrophotometer [32]. All assays were performed in triplicate. Semi-logarithmic plots were constructed from the averaged data, and the effective doses of extracts required to inhibit cell growth by 50% (IC_50_) were determined. Gliotoxin (1 μg/mL) was used as a positive control and BME as a negative control.

Values of IC_50_ of crude extracts were chosen according to the NCI screening program: extracts of IC_50_ > 30 μg/mL can be considered inactive; extracts of 10 < IC_50_ < 30μg/mL are weakly active, extracts with IC_50_ < 10 μg/mL are highly active [12].

## 4. Conclusion

A methodology for the production and extraction of bioactive hydrophilic fungal metabolites has been developed. Our model consists of fungal production by ASF and extraction by an aqueous process using cryo-crushing of mycelium and enzymatic digestion of medium by agarase. These results can be improved by evaluating different compositions of medium. The model proposed in this study will be implemented in our lab, and its application on strains from our marine fungal collection will complete the well-established screening of hydrophobic bioactive metabolites and improve the efficiency of the search for bioactive fungal metabolites by screening them over all their polarity range.

## Figures and Tables

**Table 1:** Protein and sugar contents of mycelium and medium crude extracts of the six studied marine fungal strains in ASF (agar surface fermentation) obtained by EOP (extraction by organic process) and EAP (extraction by aqueous process) and in SmF (submerged fermentation) obtained by EAP.

	
	EOP				EAP							
	
	ASF				ASF				SmF			
	
	mycelium		agar-agar		mycelium		agar-agar		mycelium		filtrate^a^	
	protein yield	sugar yield	protein yield	sugar yield	protein yield	sugar yield	protein yield	sugar yield	protein yield	sugar yield	protein yield	sugar yield
	(mg/g dw)	(mg/g dw)	(mg/mL)	(mg/mL)	(mg/g dw)	(mg/g dw)	(mg/mL)	(mg/mL)	(mg/g dw)	(mg/g dw)	(mg/mL)	(mg/mL)
***Penicillium citreonigrum***	0.78	14.25	0.07	0.25	10.67	34.19	0.55	2.13	9.52	35.41	0.36	2.27
***Phoma*sp.**	1.11	11.11	0.09	0.65	14.45	33.32	0.60	3.56	13.87	32.15	0.37	4.13
***Phoma exigua*var*exigua***	2.48	20.23	0.05	0.31	10.50	44.25	0.22	0.85	11.19	42.63	0.19	0.77
***Scopulariopsis*sp.**	4.01	36.43	0.12	0.21	16.04	51.35	0.42	1.33	16.54	50.86	0.28	1.19
***Chrysosporium queenslandicum***	5.70	37.66	0.08	0.45	21.03	55.28	0.15	2.43	23.17	53.77	0.13	2.25
***Pycnidiophora dispersa***	2.43	20.30	0.09	0.42	12.44	46.66	0.20	2.97	11.35	45.34	0.18	2.08
**average**	**2.75**	**23.33**	**0.08**	**0.38**	**14.19**	**44.18**	**0.36**	**2.21**	**14.27**	**43.36**	**0.25**	**2.12**

a expressed per mL of culture medium.

**Table 2:** Neuroactivity on Diptera larvae expressed by the MED (minimal effective dose) in mg/mL and cytotoxicity on KB cells expressed by the IC_50_ (inhibition concentration of 50% of cellular growth) in μg/mL of mycelium and medium crude extracts of the six studied marine fungal strains cultivated in ASF (agar surface fermentation) extracted by EOP (extraction by organic process) and EAP (extraction by aqueous process) and in SmF (submerged fermentation) extracted by EAP.

	
	EOP				EAP							
	
	ASF				ASF				SmF			
	mycelium		agar-agar		mycelium		agar-agar		mycelium		filtrate	
	neuroactivity	cytotoxicity	neuroactivity	cytotoxicity	neuroactivity	cytotoxicity	neuroactivity	cytotoxicity	neuroactivity	cytotoxicity	neuroactivity	cytotoxicity
	MED	IC_50_	MED	IC_50_	MED	IC_50_	MED	IC_50_	MED	IC_50_	MED	IC_50_
	mg/mL	μg/mL	mg/mL	μg/mL	mg/mL	μg/mL	mg/mL	μg/mL	mg/mL	μg/mL	mg/mL	μg/mL
***Penicillium citreonigrum***	100	-	100	-	12.5	-	100	-	50	-	-	-
***Phoma*sp.**	50	-	-	-	25	68	100	130	50	62	100	85
***Phoma exigua*var*exigua***	50	-	100	-	-	-	-	57	-	-	-	97
***Scopulariopsis*sp.**	100	-	100	-	100	-	-	44	-	-	-	48
***Chrysosporium queenslandicum***	100	-	100	-	100	-	-	-	100	-	-	-
***Pycnidiophora dispersa***	100	-	-	-	-	70	-	-	-	94	-	-

**inert medium**			-	-			-	-			-	-

The sign **–** means the absence of neuroactivity or cytotoxicity at the highest tested concentrations.

Positive control: - neuroactivity: domoic acid; MED = 50 ng/mL; - cytotoxicity: gliotoxin; IC_50_ = 0.3 μg/mL.

**Table 3:** Biomass of the six studied marine fungal strains obtained by ASF (agar surface fermentation) and SmF (submerged fermentation).

	Biomass^a^ (g dw/L)	
	ASF	SmF
***Penicillium citreonigrum***	50.0 ± 2.1	35.6 ± 1.8
***Phoma*sp.**	33.3 ± 1.7	7.1 ± 0.2
***Phoma exigua*var*exigua***	53.4 ± 2.4	6.2 ± 0.3
***Scopulariopsis*sp.**	57.9 ± 2.6	2.0 ± 0.1
***Chrysosporium queenslandicum***	5.8 ± 0.3	0.8 ± 0.3
***Pycnidiophora dispersa***	54.2 ± 1.9	31.7 ± 1.5
**average**	**42.4**	**13.9**

a expressed per liter of culture medium; results represent biomass mean ± SD (n = 3).

## Abbreviations

ASF: agar surface fermentation
EAP: extraction by aqueous process
EOP: extraction by organic process
MED: minimal effective dose
SmF: submerged fermentation
SSF: solid state fermentation
